# A Datasheet for the INSIGHT Moorfields Cornea Anterior Segment Dataset (CADMUS)

**DOI:** 10.1016/j.xops.2026.101203

**Published:** 2026-04-22

**Authors:** Shafi Balal, Zaid Alsafi, Marcello Leucci, Pearse A. Keane, Daniel Gore, Nikolas Pontikos, Bruce D. Allan

**Affiliations:** 1 Moorfields Eye Hospital NHS Foundation Trust, London, United Kingdom; 2 Institute of Ophthalmology, University College London, London, United Kingdom; 3 NIHR Moorfields Biomedical Research Centre, London, United Kingdom

**Keywords:** Anterior segment imaging, Cornea, OCT, Dataset, Artificial intelligence

## Abstract

**Purpose:**

To describe Cornea Anterior Segment Dataset from Moorfields via INSIGHT (CADMUS), a large multimodal anterior segment imaging dataset developed through the Eye and Oculomics Health Data Research Hub (INSIGHT) Health Data Research Hub to support corneal research and artificial intelligence (AI) development.

**Design:**

A dataset description.

**Participants:**

Anonymized anterior segment imaging and linked clinical metadata from 22 482 patients attending Moorfields Eye Hospital National Health Service (NHS) Foundation Trust between December 2019 and September 2024 (refreshed every 2 years).

**Methods:**

The CADMUS was derived from routine clinical care using INSIGHT infrastructure. It comprises raw Digital Imaging and Communications in Medicine MS-39 Placido images, OCT scans, grayscale photographs, derived tomography indices, and linked electronic health record metadata including demographics, diagnoses, procedures, clinic activity, refraction, visual acuity, and follow-up. Data were pseudonymized, standardized, and curated within the INSIGHT Secure Research Environment under established governance and ethical approval.

**Main Outcome Measures:**

Dataset scale, multimodal integration, longitudinal follow-up, disease representation, surgical data capture, and availability of linked imaging and clinical metadata.

**Results:**

The CADMUS includes 945 243 image acquisitions from 22 482 patients, with 278 101 tomography records, 514 524 clinic visits, and 40 471 surgical records from 12 079 patients. Additional data include 52 463 refraction measurements (13 235 patients) and 543 902 visual acuity records (22 368 patients). Longitudinal follow-up is present in 96.1% of patients (mean 6.1 visits). The dataset captures diverse anterior segment pathology, including keratoconus (42 194 records), nuclear cataract (11 800), corneal scar (3,430), Fuchs’ dystrophy (2652), and corneal decompensation (1264). Common procedures include phacoemulsification (8209), corneal cross-linking (7115), Descemet membrane endothelial keratoplasty (1739), penetrating keratoplasty (1308), and laser superficial keratectomy/phototherapeutic keratectomy (1189).

**Conclusions:**

The CADMUS is a large, longitudinal, multimodal anterior segment dataset linking imaging with detailed clinical metadata in a secure NHS environment. It provides a robust platform for reproducible research, longitudinal modeling, surgical outcomes analysis, and AI development in anterior segment disease.

**Financial Disclosures:**

Proprietary or commercial disclosure may be found in the Footnotes and Disclosures at the end of this article.

Corneal opacities cause 3.2% of global blindness and 1.3% of moderate-to-severe vision impairment, ranking among the top 5 causes of blindness worldwide.^1^ These conditions disproportionately impact younger populations and regions with limited health care resources, creating an urgent need for improved diagnostic and monitoring capabilities. Early diagnosis and timely intervention are critical, as many anterior segment pathologies can now be stabilized or reversed through treatments such as corneal cross-linking, lamellar keratoplasty, or minimally invasive laser refractive procedures.^2^

Modern imaging modalities—including high-resolution anterior-segment OCT, Scheimpflug tomography, and Placido-disc topography—have revolutionized our ability to quantify corneal morphology, biomechanics, and disease progression.^3^ Among these, the MS-39 OCT tomographer (CSO) combines Placido-based curvature mapping with swept-source OCT cross-sectional imaging, providing accurate 3-dimensional reconstruction of both anterior and posterior corneal surfaces.^4^

Despite advances in corneal imaging technology and artificial intelligence (AI) applications in ophthalmology, progress in corneal disease has been limited by the scarcity of well-curated, accessible anterior segment image datasets.^5^ A 2021 systematic review found that <10% of ophthalmic imaging datasets addressed the anterior segment.^6^ More recently, a review of publicly available anterior segment image datasets found that most were relatively small, heavily skewed toward normal eyes, and lacked standardized metadata or broad disease representation.^7^

The Eye and Oculomics Health Data Research Hub (INSIGHT) led by Moorfields Eye Hospital National Health Service (NHS) Foundation Trust (Moorfields), has established the world's largest bioresource of ophthalmic images.^8^ Containing >30 million eye images, and linking to key labeling fields in electronic health care records, INSIGHT provides secure access to large-scale anonymized clinical data for approved research. Enabled by INSIGHT’s infrastructure, the Cornea Anterior Segment Dataset from Moorfields via INSIGHT (CADMUS) dataset, developed from raw MS-39 images, processed data outputs, and associated labeling fields from the electronic health care record acquired in the course of routine clinical care at Moorfields, is a large, multimodal anterior segment imaging dataset. The CADMUS was designed to address problems with existing publicly accessible anterior segment imaging datasets including small size, lack of data format standardization, inadequate data labeling, absence of follow-up data, and a poorly defined framework for data access. Researchers seeking to access to the CADMUS dataset can apply through INSIGHT’s governance framework which includes the Data Use Application process.

To enhance transparency and facilitate appropriate research use, this datasheet provides researchers with information about the composition of the CADMUS dataset, collection processes, preprocessing methods, potential uses, and distribution mechanisms.

## Dataset Motivation

The clinical and research rationale underpinning CADMUS is multifaceted. From a clinical perspective, diseases of the anterior segment—including keratoconus and other corneal ectasias, endothelial dystrophies, postrefractive and postcataract surgery complications, and anterior chamber angle abnormalities—represent a significant health care burden in terms of visual morbidity, treatment complexity, and long-term outcomes.^9^ Advances in imaging (e.g., anterior-segment OCT) have improved our ability to detect structural changes, monitor progression, and tailor interventions.^4^ Despite these advances, challenges remain. Decision tools for early disease detection, prediction of progression risk, robust tracking of longitudinal change, and tools designed to work with the integration of multimodal imaging and detailed metadata remain under developed.^10^ From a research perspective, these gaps translate into unmet needs in biomarker discovery, surgical outcome prediction, and algorithmic decision support in corneal and anterior segment practice. The CADMUS dataset project was motivated by the need to bridge the gap between clinical needs and research capability by assembling a rich, longitudinal, well-curated image-metadata resource.

The creation of large, well-annotated imaging datasets is a critical enabler of technological innovation. In the domain of AI, machine learning and deep learning approaches are particularly well suited to image-based pattern recognition and prognostic modeling. Artificial intelligence modeling requires adequately sized, high-quality, labeled datasets for both training and validation. Lack of access to high-quality imaging datasets has limited the generalizability of AI models of anterior segment disease, undermined reproducibility and slowed translation of new models into a clinical practice setting. The CADMUS was designed to enable a broad spectrum of use-cases: from development and external validation of deep learning models for anterior segment disease detection and progression prediction, to epidemiological and longitudinal analyses of corneal morphometry, surgical outcomes research, and educational benchmarking. By providing open and accessible data within a secure governance framework, CADMUS seeks to accelerate innovation and ultimately enhance patient care in corneal and anterior segment disease.

## Dataset Composition

The CADMUS dataset comprises raw Digital Imaging and Communications in Medicine (DICOM) MS-39 Placido, sectional OCT and grayscale photographic images, derived quantitative indices relevant to anterior segment disease, and labeling data from the electronic health care record describing demographic details, interventions, and follow-up intervals, and metadata collected from patients attending Moorfields. The dataset has been curated through INSIGHT and structured for research use within the INSIGHT Secure Research Environment. All data are collected during routine clinical care, anonymized, and prepared for research use through the INSIGHT data governance framework. Details of the CADMUS data dictionary are provided in the Supplementary Information below. Compilation of the data set adhered to the tenets of the Declaration of Helsinki and has approval from the relevant institutional review board and ethical approval from the Research Ethics Service (Research Ethics Committee reference: 20/WS/0087).

## Imaging Modalities

**Figure 1:**
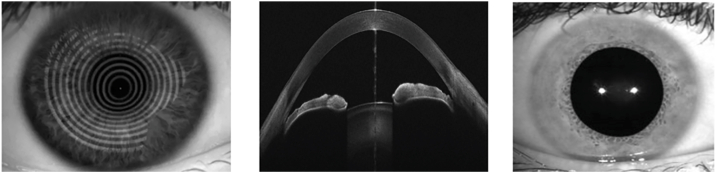
Representative MS-39 imaging output showing Placido-disc–based anterior corneal topography, radial swept-source OCT cross-section, and external eye photograph.

The CADMUS is based primarily on the MS-39 anterior-segment OCT—providing high-resolution cross-sectional and tomographic scans. It comprises radial OCT cross-sections, Placido-based corneal topography, and external eye photographs (Fig 1). The MS-39 employs a combination of spectral-domain OCT and Placido-disk corneal topography to measure the anterior segment of the eye. The scanning process, which takes around 1 second, begins after autocalibration and is recorded using the Phoenix software (version 4.1.3). In the default acquisition mode, the MS-39 captures a single Placido top-view image and a set of 25 or 12 radial spectral-domain OCT scans. These scans are performed using a wavelength of 840 μm, resulting in an axial resolution of 3.5 μm and a transverse resolution of 35 μm. The width of each OCT section is 10 or 16.0 mm, and the maximum depth is 7 mm. Each OCT section is comprised of 1024 A-scans. Provided there is adequate corneal surface coverage, the Placido image is used to derive processed numeric outputs describing anterior corneal curvature and for wavefront analysis. A proprietary method is employed to merge the anterior surface data obtained from the Placido image with that from the spectral-domain OCT scans to obtain elevation maps. Other measures, including segmental pachymetry and posterior corneal curvature, are derived exclusively from the OCT data.^11^

## Dataset Characteristics

The dataset contains 945 243 image acquisitions from 22 482 unique patients. Each record is linked across imaging modalities and clinical encounters using a pseudonymized patient identifier. The dataset spans the period from December 2019 to September 2024, with approximately 96.1% of patients having multiple follow-up visits, with a mean of 6.1 visits per patient, allowing for longitudinal modeling and disease trajectory analyses.

## Demographic Characteristics

**Figure 2:**
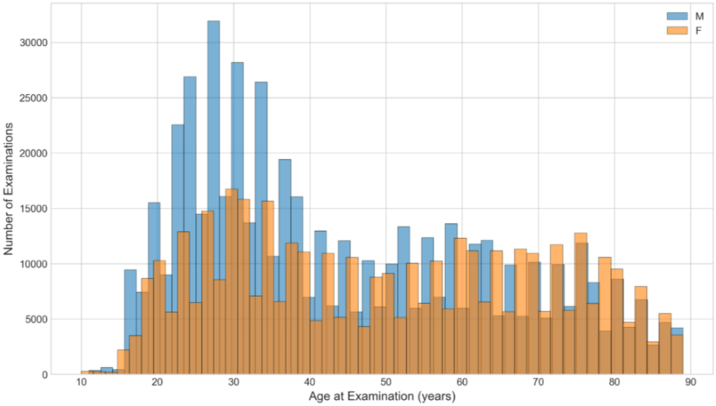
Age distribution of patients in the CADMUS dataset stratified by sex. Male patients (n = 12 168; 54.1%) shown in blue; female patients (n = 10 314; 45.9%) shown in orange. Median age 43 years (IQR 29–64). CADMUS = Cornea Anterior Segment Dataset from Moorfields via INSIGHT; IQR = interquartile range.

**Figure 3:**
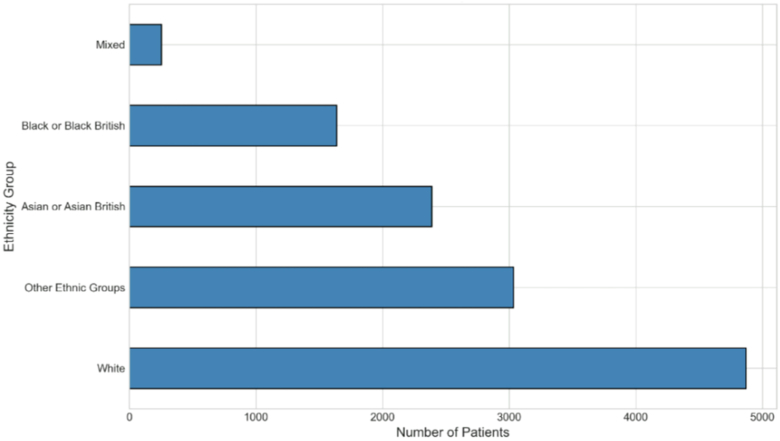
Self-reported ethnicity distribution of unique patients (N = 11 482) in the CADMUS dataset. CADMUS = Cornea Anterior Segment Dataset from Moorfields via INSIGHT.

The cohort reflects the demographic diversity of the Moorfields patient population, with broad representation across ethnicities and age groups typical of London and the UK catchment area (Table 1; Figs 2 and 3).

**Table 1:** Summary Characteristics of the CADMUS Dataset Including Patient Demographics, Imaging Data, Clinical Diagnoses, and Surgical Procedures

Category	Metric	Value
Dataset overview	Total unique patients	22 482
	Total image acquisitions	945 243
	Date range	December 2019 – September 2024
	Tomography tabular data (rows)	278 101
Demographics	Male	54.1%
	Female	45.9%
	Age, mean (SD)	47.0 (20.1) years
	Age, median (IQR)	43.0 (29.0–64.0) years
	Age range	10–89 years
Ethnicity	White	40.0%
	Other Ethnic Groups	24.9%
	Asian or Asian British	19.6%
	Black or Black British	13.4%
	Mixed	2.1%
Imaging	Corneal iris photography	320 651
	Keratoscope topography	316 141
	OCT cross-sections	308 451
	Right eye images	474 720 (50.2%)
	Left eye images	470 523 (49.8%)
Longitudinal data	Patients with multiple visits	5676 (96.1%)
	Mean visits per patient	6.1
	Maximum visits	22
Diagnoses	Total diagnosis records	140 716
	Patients with diagnoses	17 966
	Keratoconus records	42 194
Operations	Total operation records	40 471
	Patients with operations	12 079
Clinical measures	Visual acuity measurements	543 902
	Refraction readings	52 463
	Medication records	567 684
	Appointment records	514 524

CADMUS = Cornea Anterior Segment Dataset from Moorfields via INSIGHT; IQR = interquartile range; SD = standard deviation.

## Diagnosis Distribution

**Figure 4:**
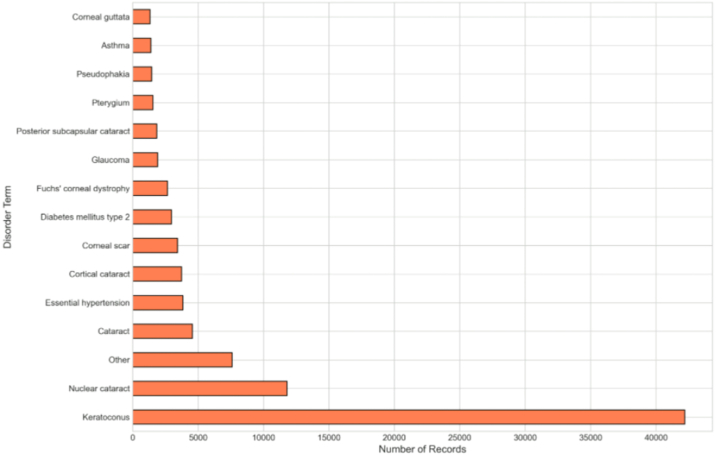
Top 15 diagnoses by frequency in the CADMUS dataset. Keratoconus represents the most prevalent diagnosis (42 194 records). CADMUS = Cornea Anterior Segment Dataset from Moorfields via INSIGHT.

The dataset captures a broad spectrum of anterior segment pathology (Fig 4). The most prevalent diagnosis is keratoconus (42 194 records), reflecting the dataset's foundation in Moorfield’s early keratoconus clinic. Other common diagnoses include nuclear cataract (11 800 records), corneal scar (3430 records), Fuchs' corneal dystrophy (2652 records), and corneal decompensation (1264 records). Systemic comorbidities are also captured, including essential hypertension (3833 records), type 2 diabetes mellitus (2960 records), and hypercholesterolemia (1274 records).

## Surgical Procedures

**Figure 5:**
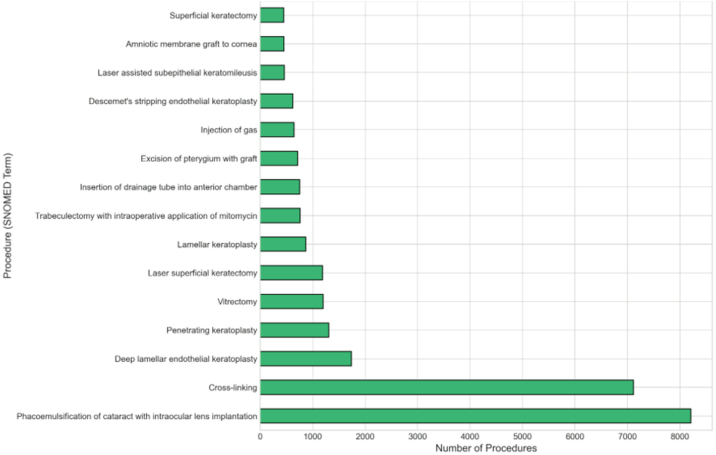
Distribution of surgical procedures (N = 40 471) showing the top 15 procedure types by SNOMED terminology. SNOMED = Systematized Nomenclature of Medicine.

A total of 40 471 operation records from 12 079 patients are recorded (Fig 5). The most common procedures include phacoemulsification cataract surgery with intraocular lens implantation (8209 procedures), corneal cross-linking (7115 procedures), deep lamellar endothelial keratoplasty (Descemet membrane endothelial keratoplasty; 1739 procedures), penetrating keratoplasty (1308 procedures), and laser superficial keratectomy/phototherapeutic keratectomy (1189 procedures). Additional keratoplasty variants include anterior lamellar keratoplasty (870 procedures) and Descemet's stripping automated endothelial keratoplasty (623 procedures).

## Clinical Sites

Operations were performed across multiple sites within the Moorfields network. City Road (the principal tertiary referral site) accounts for 30 629 procedures (75.7%), with additional procedures performed at St George's Hospital (3713), Croydon University Hospital (1733), St Ann's Hospital (895), and Ealing Hospital (825), among other satellite locations.

## Clinic Distribution

The 514 524 appointments span multiple subspecialty clinics, with the largest volumes in external disease (EX; 174 788), contact lens (CL; 70 632), glaucoma (GL; 57 361), keratoconus (37 796), and refractive (RF; 21 594) clinics.

## Tomography Tabular Data

Tomography data include 278 101 rows of tabular data consisting of MS-39-derived metrics. Each row includes patient and examination identifiers, laterality, event date and time, age at examination, and device-specific resolution parameters. Keratometry values are captured for both anterior and posterior corneal surfaces, including maximum keratometry and flat/steep meridian measurements. The dataset includes MS-39 classifier probability scores for keratoconus, subclinical keratoconus, normal, myopic postoperative, and abnormal corneal states. Acquisition quality metrics document OCT section coverage, Placido coverage, Placido decentering, and maximum Placido ring captured. Anatomical measurements include pupil center coordinates and thinnest point locations for both epithelium and stroma. Summary indices comprise corneal volume, central corneal thickness, anterior chamber depth, and minimum thickness with its centroid location. Wavefront aberrometry data are provided for both anterior surface and total corneal optical path difference, including higher-order aberration root mean square, analysis diameter, corneal power, cylinder and axis, and individual Zernike coefficients (piston, tilt, astigmatism, defocus, trefoil, coma, quadrafoil, secondary astigmatism, and spherical aberration) with corresponding axes where applicable.

## Refraction Data

Refraction readings are available for 13 235 patients (52 463 measurements). Mean spherical equivalent is –0.30 D (standard deviation: 3.68), with mean cylinder of –2.58 D (standard deviation: 2.52), skewed by the high prevalence of keratoconus in this population.

## Visual Acuity

Visual acuity measurements (543 902 records; 22 368 patients) were available via multiple methods: pinhole (183 247), spectacle-corrected (154 323), unaided (153 258), contact lens (28 213), and formal refraction (24 617). Measurements were recorded predominantly in Snellen meter (378 310; 69.6%) and logarithm of the minimum angle of resolution (119 605; 22.0%) notation.

## Preprocessing Pipeline for Image and Metadata

Images were securely exported in DICOM format and processed within the INSIGHT cloud-based environment. Imaging data were linked to relevant clinical information from the Moorfields electronic health record using pseudonymous identifiers. Preprocessing focused on preparing the data for research use and included standardization of file formats, data types, and units to ensure consistency across the dataset.

## Quality Control and Validation Steps

All images were included with appropriate quality metrics to allow researchers to apply their own exclusion criteria and quality control checks. Clinical metadata were reviewed for completeness and internal consistency and to identify missing or implausible values. Where necessary, targeted manual review was undertaken to resolve inconsistencies before dataset release.

## Anonymization and Data Standardization

The dataset is fully anonymized using established INSIGHT cloud-based data governance infrastructure and processes. Patient records are pseudonymized at the earliest stage using a secret one-way cryptographic hash and irreversibly anonymized before researcher access. Secure export of images is enabled within the imaging device software, and extraction of relevant associated clinical data from the Moorfields electronic health record system are undertaken under approved governance arrangements within the INSIGHT Hub Program.

## Dataset Versioning

This datasheet summarizes the first version of the dataset release, comprising data collected up to March 2025. The dataset is intended to be refreshed periodically with additional data collected from existing sites. New records will be matched to previously collected data using consistent deidentification methods, ensuring that each unique subject retains the same identifier.

## Ethical and Governance Framework

The CADMUS dataset operates within the robust ethical and governance structure established by INSIGHT, ensuring the responsible use of NHS patient data in compliance with Health Research Authority research ethics committee approval and all UK data protection legislation. Ethical approval for the INSIGHT Research Database was granted by the West of Scotland Research Ethics Service (Research Ethics Committee reference: 20/WS/0087), which covers the collection, curation, and research use of anonymized patient data from Moorfields Eye Hospital NHS Foundation Trust and other participating data collection centers.^12^ All data are processed and deidentified in accordance with the tenets of the Declaration of Helsinki and both General Data Protection Regulation and UK General Data Protection Regulation principles, and guidance from the UK Information Commissioner’s Office. In accordance with General Data Protection Regulation Article 9.2 exemptions, research ethics committee approval allows retrospective inclusion of routinely collected clinical data without explicit individual patient consent, provided that no identifiable information is accessible to researchers.

Patients’ rights to control the use of their data are protected through the NHS National Data Opt-Out program, which permits individuals to decline the use of their confidential information for research and planning purposes beyond their direct care. This mechanism is fully implemented within the INSIGHT data collection mechanism, ensuring that any patient who has opted out is excluded from data extraction and processing prior to anonymization. The INSIGHT employs a layered governance model aligned with the “Five Safes” framework (Safe Projects, Safe People, Safe Data, Safe Settings, and Safe Outputs).^13^

## Dataset Uses

The CADMUS dataset builds on a small but growing body of ophthalmic research that has harnessed large-scale anterior segment imaging and clinical data for both descriptive and predictive modeling purposes. For example, our group utilized repeated corneal tomography scans in a keratoconus population to map device-specific precision limits for the MS-39 throughout the range of disease severity and to examine the effect of averaging repeated measures from a single timepoint. Precise mapping of precision limits for MS-39 indices in keratoconus was then used as a rational basis for the definition of disease progression, and ground truth labeling in downstream modeling.^4^ In another study, using an extract from the large volume of labeled images in CADMUS, we trained deep learning models to predict age and biological sex, illustrating the way in which routinely collected anterior segment image data contains biosignals that are not readily apparent to the human observer.^14^ These examples demonstrate how richly annotated anterior segment datasets can underpin both classical statistical studies of disease behavior and emergent AI-driven workflows.

Looking ahead, the CADMUS dataset offers considerable potential for future research across multiple domains. First, longitudinal imaging allows analyses of disease progression. Second, because CADMUS includes both image and clinical metadata (e.g., anterior segment tomography, surgery details, demographics, and comorbidities), it facilitates multimodal predictive modeling: for example, which eyes will require surgical intervention or respond to treatment. Third, CADMUS can support the validation and generalization of AI/machine learning models developed elsewhere: the dataset can act as an external test bed for algorithms trained in 1 center or device. Fourth, given the large volume and diversity of data, research on health-equity and demographic bias (e.g., device performance across age, ethnicity, or ocular comorbidities) becomes feasible. In short, CADMUS is explicitly designed to serve as a broad platform for both the descriptive epidemiology of anterior segment disease and the development, validation, and deployment of future ophthalmic imaging innovations.

## Access

The CADMUS dataset is accessible to approved academic, industry, and charity researchers via the INSIGHT Hub's established data-access infrastructure. Interested researchers first consult the INSIGHT portal to discover available datasets and submit an initial data-access enquiry, followed by a Data Use Application outlining their institutional affiliation, project aims, required fields (including imaging types, clinical metadata, and follow-up timepoints) and demonstrating clear public and patient benefit. The application then undergoes a rigorous 3-stage review: (1) due-diligence checks by the INSIGHT team (Safe People and Safe Projects); (2) independent scrutiny by the INSIGHT Data Trust Advisory Board (DataTAB), which evaluates alignment with the Five Safes framework and public benefit; and (3) legal/contractual approval by the NHS data controller (Moorfields Eye Hospital NHS Foundation Trust) before a Data Use Agreement is executed. Once approved, access is provisioned within the INSIGHT Secure Research Environment. This framework ensures that CADMUS, as part of the wider INSIGHT infrastructure, supports responsible data-driven innovation in ophthalmology while maintaining the highest ethical standards for privacy, security, and accountability. Enquiries can be made through the following link: https://www.insight.hdrhub.org/.

The INSIGHT Secure Research Environment is a virtualized secure computing environment that hosts anonymized/pseudonymized images and metadata and restricts data export to approved outputs only. Within the secure research environment, researchers are provided a virtual machine with preapproved software tools and analytic environment; raw data cannot leave the environment unless an export request is submitted and audited by the data controller. While licensing terms may vary depending on academic versus commercial use, INSIGHT emphasizes use for patient-benefit research, anonymized datasets, and the requirement for approved outputs to be publicly registered in the INSIGHT Data Use Register. In terms of formats, data are delivered in standard interoperable formats (e.g., DICOM or proprietary imaging modality formats for anterior-segment scans, plus associated CSV tables of clinical metadata) and are accessible within the secure research environment; external download of raw DICOM files is subject to specific justification and contractual clarity (where permitted). Users should reference the CADMUS data dictionary (and accompanying documentation) for the exact formats and fields.

## Strengths and Limitations

The CADMUS dataset represents one of the largest and most detailed resources of routinely collected anterior segment and corneal data within the NHS. Its principal strengths lie in its scale, longitudinal coverage, and multimodal nature—linking structured clinical data with high-resolution anterior segment imaging modalities such as corneal topography, tomography, and slit-lamp photography. Because it is derived from the clinical records of Moorfields Eye Hospital, a tertiary referral center serving a demographically diverse population, the dataset captures a broad spectrum of corneal disease, surgical indications, and postoperative outcomes. This diversity enhances generalizability for both clinical research and model development. Furthermore, alignment with the INSIGHT governance framework ensures rigorous data quality, reproducibility, and ethical oversight, making it a trustworthy foundation for translational studies and AI validation. The continuous data-pipelining model also ensures that CADMUS remains up to date, supporting long-term monitoring, and evaluation of changes in corneal care delivery across the NHS.

Nevertheless, there are inherent limitations associated with data routinely collected in a clinical setting. Some clinical fields may be incomplete or inconsistently recorded across time and devices, especially during periods of system transition or equipment upgrade. Imaging protocols and calibration parameters may vary between devices and operators, introducing potential heterogeneity that researchers must address during analysis. As with all retrospective datasets, CADMUS does not include explicit patient-reported outcomes or prospective consent, and data linkage beyond Moorfields Eye Hospital may be restricted to approved INSIGHT pipelines. Finally, while the dataset provides exceptional depth for corneal and anterior segment disease, generalization for models trained on the CADMUS population when applied to distinct patient populations requires adequate examination for each use case. Despite these constraints, the CADMUS dataset should help to provide an opportunity to advance the understanding of anterior segment disease and to develop and test AI tools in a secure, ethically governed NHS environment.

## Summary and Future Directions

The CADMUS provides a uniquely comprehensive and ethically governed resource for the study of corneal and anterior segment disease. By integrating multimodal imaging, longitudinal clinical data, and surgical outcomes within a single anonymized NHS dataset, CADMUS enables reproducible research into disease mechanisms, prognostic modeling, and AI-driven diagnostics. Future developments will focus on expanding data coverage to include emerging 3-dimensional anterior segment imaging modalities, incorporation of AI-derived annotations, and linkage with wider systemic datasets to explore ocular–systemic relationships. Continued updates through INSIGHT’s “evergreen” data pipeline will ensure that CADMUS remains current and representative of clinical practice. The dataset is intended as a shared national resource, and the authors invite collaboration from the academic, clinical, and industry communities to maximize its translational impact for patient benefit.

## References

[1] Wang E.Y., Kong X., Wolle M. (2023). Global trends in blindness and vision impairment resulting from corneal opacity 1984–2020: a meta-analysis. Ophthalmology.

[2] Saldanha I.J., Lindsley K.B., Lum F. (2019). Reliability of the evidence addressing treatment of corneal diseases: a summary of systematic reviews. JAMA Ophthalmol.

[3] Han S.B., Liu Y.-C., Mohamed-Noriega K., Mehta J.S. (2021). Advances in imaging technology of anterior segment of the eye. J Ophthalmol.

[4] Balal S., Cai Y., Kandakji M.L. (2025). Establishing the ground truth for keratoconus progression: combining repeated measures and adapting precision limits to disease severity in tomography. J Cataract Refract Surg.

[5] Xu Z., Xu J., Shi C. (2023). Artificial intelligence for anterior segment diseases: a review of potential developments and clinical applications. Ophthalmol Ther.

[6] Khan S.M., Liu X., Nath S. (2021). A global review of publicly available datasets for ophthalmological imaging: barriers to access, usability, and generalisability. Lancet Digital Health.

[7] Niestrata M., Radia M., Jackson J., Allan B. (2024). A global review of publicly available image datasets for the anterior segment of the eye. J Cataract Refract Surg.

[8] INSIGHT The health data research hub for eye health. https://www.insight.hdrhub.org/.

[9] Alawa K.A., Sales C.S. (2021). Alleviating an increasingly burdened healthcare system with telemedicine: anterior segment. Ophthalmol Ther.

[10] Maile H., Li J.-P.O., Gore D. (2021). Machine learning algorithms to detect subclinical keratoconus: systematic review. JMIR Med Inform.

[11] Venkataraman A.P., Domínguez-Vicent A., Selin P. (2022). Precision of a new SS-OCT biometer to measure anterior segment parameters and agreement with 3 instruments with different measurement principles. J Cataract Refract Surg.

[12] Stagg H.R., Jones J., Bickler G., Abubakar I. (2012). Poor uptake of primary healthcare registration among recent entrants to the UK: a retrospective cohort study. BMJ open.

[13] Ritchie F. (2017). The ‘five safes’: a framework for planning, designing and evaluating data access solutions. Data Policy.

[14] Balal S., Cox L., Khan A. (2025). Investigating the capability of deep learning models to predict age and biological sex from anterior segment ophthalmic imaging: a multi-centre retrospective study. BMJ open.

